# Integrating Paleodistribution Models and Phylogeography in the Grass-Cutting Ant *Acromyrmex striatus* (Hymenoptera: Formicidae) in Southern Lowlands of South America

**DOI:** 10.1371/journal.pone.0146734

**Published:** 2016-01-06

**Authors:** Maykon Passos Cristiano, Danon Clemes Cardoso, Tânia Maria Fernandes-Salomão, Jürgen Heinze

**Affiliations:** 1 Departamento de Biologia Geral, Universidade Federal de Viçosa, Av. Peter Henry Rolfs, s/n, Viçosa, Minas Gerais, 36570–000, Brazil; 2 Departamento de Biodiversidade, Evolução e Meio Ambiente/ICEB, Universidade Federal de Ouro Preto, Campus Morro do Cruzeiro, Ouro Preto, Minas Gerais, 35400–000, Brazil; 3 Departamento de Genética, Setor de Ciências Biológicas, Universidade Federal do Paraná, Rua Francisco H. dos Santos, 100, Jardim das Américas, Curitiba, Paraná, 81530–000, Brazil; 4 Zoology / Evolutionary Biology, Universitätstrasse 31, Universität Regensburg, 93040, Regensburg, Deutschland; University of Innsbruck, AUSTRIA

## Abstract

Past climate changes often have influenced the present distribution and intraspecific genetic diversity of organisms. The objective of this study was to investigate the phylogeography and historical demography of populations of *Acromyrmex striatus* (Roger, 1863), a leaf-cutting ant species restricted to the open plains of South America. Additionally, we modeled the distribution of this species to predict its contemporary and historic habitat. From the partial sequences of the mitochondrial gene cytochrome oxidase I of 128 *A*. *striatus* workers from 38 locations we estimated genetic diversity and inferred historical demography, divergence time, and population structure. The potential distribution areas of *A*. *striatus* for current and quaternary weather conditions were modeled using the maximum entropy algorithm. We identified a total of 58 haplotypes, divided into five main haplogroups. The analysis of molecular variance (AMOVA) revealed that the largest proportion of genetic variation is found among the groups of populations. Paleodistribution models suggest that the potential habitat of *A*. *striatus* may have decreased during the Last Interglacial Period (LIG) and expanded during the Last Maximum Glacial (LGM). Overall, the past potential distribution recovered by the model comprises the current potential distribution of the species. The general structuring pattern observed was consistent with isolation by distance, suggesting a balance between gene flow and drift. Analysis of historical demography showed that populations of *A*. *striatus* had remained constant throughout its evolutionary history. Although fluctuations in the area of their potential historic habitat occurred during quaternary climate changes, populations of *A*. *striatus* are strongly structured geographically. However, explicit barriers to gene flow have not been identified. These findings closely match those in *Mycetophylax simplex*, another ant species that in some areas occurs in sympatry with *A*. *striatus*. Ecophysiological traits of this species and isolation by distance may together have shaped the phylogeographic pattern.

## Introduction

Climatic oscillations during the Quaternary Period have a strong effect on the genetic diversity and distribution of extant species [1–4]. Increased aridity and decreased temperatures during the glacial led to a fragmentation of tropical forests, and forest species became restricted to stable wetland refuges [5–9]. In South America, phylogenetic studies have revealed a high genetic diversity and endemism in the fauna of tropical forests, especially of the Amazon and Atlantic Forest biomes [8, 10, 11]. This, together with paleoclimate models, indicates that many animals persisted during the glacial maxima in forest fragments north of their current range and subsequently spread south [3, 8].

However, for southern Brazil, several studies have pointed out ambiguities in the predictions from these models [12, 13]. It appears that the cool and dry weather during the glaciations allowed grassland ecosystems and open vegetation environments to expand over much of South America [14]. Fossil pollen suggests that subtropical grasslands increased their range to the north by at least 750 km (to latitude 20°S) [14, 15]. Grasslands today occur in patches in the southern highlands of Brazil (especially in Santa Catarina), where they are maintained by a climate with long periods of drought and low rainfall [14]. Subtropical grasslands, known as “campos sulinos” or “Pampas,” can also be found in the plains in southern Brazil in the state of Rio Grande do Sul, where they connect with the Pampas of Argentina and Uruguay [16]. Taxa from grassland habitats likely responded to climatic oscillations in the Quaternary Period in a different way than forest species [17–21]. Despite the potential for better understanding the patterns of diversity and distribution, only few studies have been conducted with such organisms and the observed patterns are still controversial [22–24].

Ants are an important component of all terrestrial ecosystems, but so far little attention has been given to the phylogeography of South American taxa [24]. The leaf-cutting ant *Acromyrmex striatus* (Roger, 1863) is restricted to grassland habitats in the temperate zones of South America (above 30°). This makes it an excellent model to evaluate the influence of historical climate fluctuations on the origin and evolutionary dynamics of plains associated with open vegetation in southern South America. *A*. *striatus* is common in coastal sandbanks (restinga) and sandy soils throughout the Pampas (including the *campos sulinos*), the Brazilian southern coast in the states of Santa Catarina and Rio Grande do Sul, parts of the Chaco in Argentina, and the extreme south of Paraguay [25]. Its nest is peculiar because workers clean the soil surface above the fungus chambers from all vegetation and dead plant material [26, 27]. In addition, *A*. *striatus* is an intriguing species with regards to taxonomic, phylogenetic, and cytogenetic aspects. Cristiano et al. (2013) indicated that *A*. *striatus* has the same chromosome number as the genus *Atta*, but in a phylogeny it clusters with neither its congeners nor *Atta*, but forms a sister group to the other leaf-cutting ants evaluated [28].

To better understand the evolutionary history of *A*. *striatus* (Roger, 1863) and to get insights into the history of South American plains we analyzed the phylogeography and population genetics of this species across much of its distribution. Our goal was to determine the genetic structure of its populations and the geographical patterns of genetic variation and to use these data to investigate how the glacial and interglacial periods have influenced the distribution in this species. In addition, we estimated the current potential distribution area and the historical potential area of *A*. *striatus* using paleoclimate models to allow a more detailed evaluation of the demographic history and phylogeographical patterns.

## Materials and Methods

### Collection of samples

**Fig 1:**
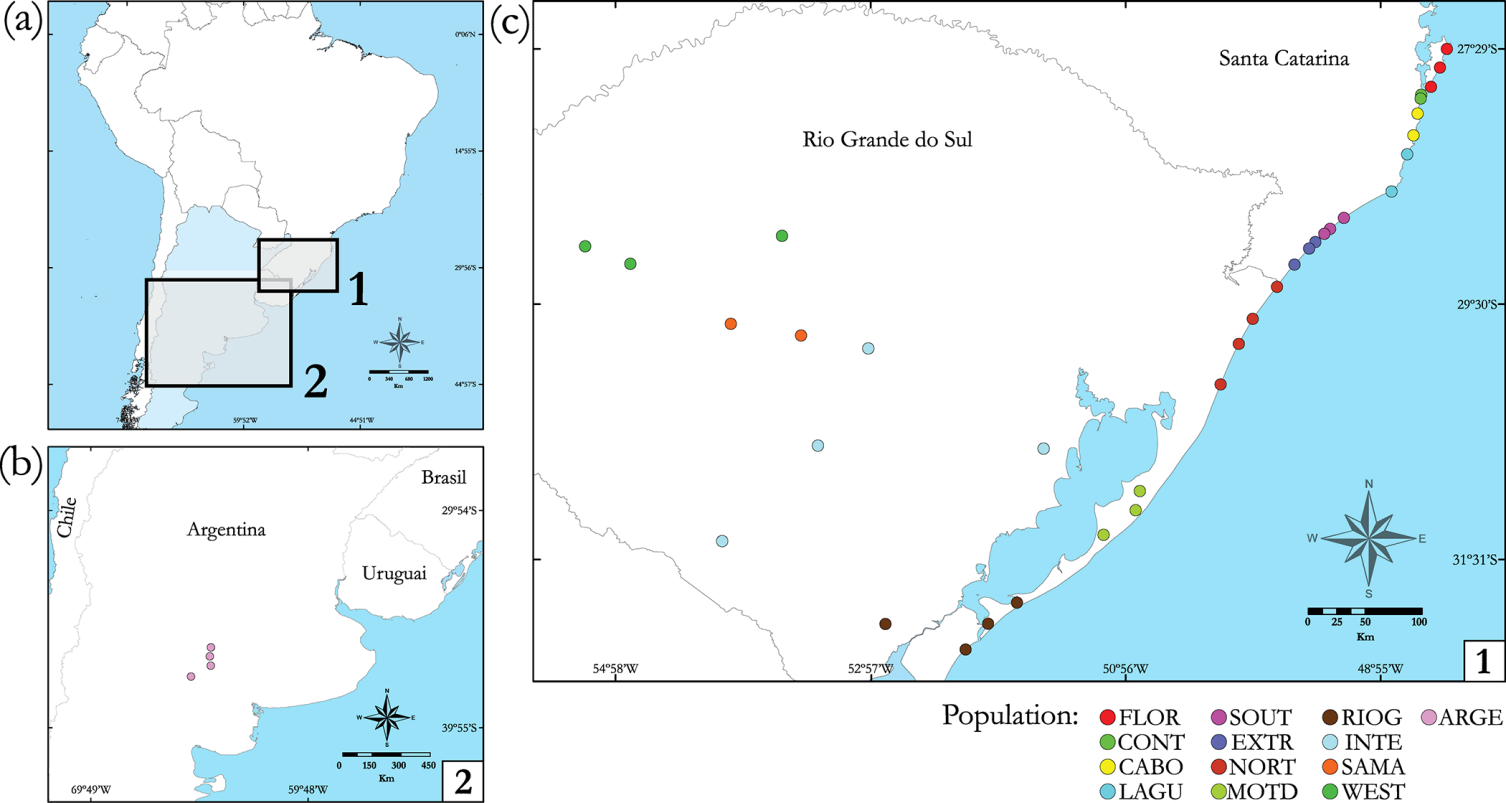
Map of South America with the geographic distribution of *Acromyrmex striatus* populations sampled. (a) Map of the South American continent, (b) population collected in Argentina and (c) populations collected in Brazil. Each collection site is represented by a point, and the colors of the points indicate each population. These same colors used to represent each population were also present in the haplotype network.

A total of 128 colonies of *Acromyrmex striatus* were collected at 38 sites in Brazil and Argentina (Table 1 and Fig 1). The specific permission for collections in Brazil (SISBio26441-1) was authorized by the Instituto Chico Mendes de Conservação da Biodiversidade (ICMBio). Samples from Argentina were kindly provided by Dr. Stela Quirán. These 38 sampling sites were pooled into 13 populations to ensure reliable estimates of regional differentiation and diversity and to allow appropriate statistical analysis. Thus, geographically and ecologically close locations (neighboring plains and/or plains inside large rivers basins) were pooled in order to obtain populations with sample sizes with of at least eight colonies. In Table 1, sampling localities belonging to the same population are shown.

**Table 1:** Specimens of *Acromyrmex striatus* sampled for phylogeographic analysis. Sampling sites, geographic coordinates (S = South, W = West), number of individuals and estimated haplotypes from the COI gene followed by the number of individuals (n). Samples collected in the states of Rio Grande do Sul and Santa Catarina are represented by RS and SC, respectively.

Country	Population	Localities (State)	Coordinates		Haplotypes (n)
			S	W	
Brazil	FLOR	Moçambique (SC)	27.48	48.38	1(4)
		Joaquina (SC)	27.63	48.45	1(1)
		Pantano do Sul (SC)	27.78	48.52	1(4)
	CONT	Pinheira (SC)	27.87	48.60	1(2)
		Sonho (SC)	27.84	48.59	1(2), 12(2)
	CABO	Garopaba (SC)	27.99	48.63	1(2), 11(2), 12(1)
		Ibiraquera (SC)	28.16	48.65	1(5)
	LAGU	Laguna (SC)	28.60	48.83	1(3), 4(1), 5(1)
		Itapirubá (SC)	28.31	48.70	1(3), 9(1), 10(1)
	SOUT	Rincão (SC)	28.81	49.20	6(1), 7(1), 8(1)
		Ilhas (SC)	28.90	49.32	1(2)
		Araranguá (SC)	28.93	49.35	1(3), 2(1)
	EXTR	Arroio do Silva (SC)	29.02	49.44	1(3), 2(1), 3(1),
		Gaivota (SC)	29.18	49.60	1(2), 3(1)
	NORT	Torres (RS)	29.36	49.74	13(1), 14(1)
		Curumim (RS)	29.62	49.93	15(1)
		Xangrilá (RS)	29.82	50.04	16(2), 17(1), 18(1)
		Cidreira (RS)	30.13	50.18	15(1), 19(1)
	MOTD	Mostardas (RS)	31.13	50.83	20(1), 21(2), 22(1), 23(1)
		Tavares (RS)	31.32	51.11	21(2), 23(1), 26(1), 27(1)
		São Simão (RS)	30.93	50.76	24(1), 25(1)
	RIOG	São José do Norte (RS)	32.02	52.03	28(2)
		Estreito (RS)	31.85	51.79	29(1), 30(1), 31(2)
		Cassino (RS)	32.11	52.17	32(1), 33(2), 34(1)
		Pedro Osório (RS)	32.02	52.83	47(2), 48(1), 49(1)
	INTE	Bagé (RS)	31.36	54.11	35(1), 50(2)
		Cachoeira do Sul (RS)	29.84	52.96	41(1), 44(1), 45(1)
		Caçapava do Sul (RS)	30.61	53.36	32(1), 33(2), 34(1)
		Tapes (RS)	30.65	51.56	51(1), 52(1)
	SAMA	Santa Maria (RS)	29.65	54.05	35(1), 36(1), 37(3)
		Restinga Seca (RS)	29.76	53.49	37(1), 38(1), 39(1), 40(1)
	WEST	Unistalda (RS)	29.03	55.21	35(1), 41(2), 42(1)
		Cruz Alta (RS)	28.95	53.64	41(3), 43(1)
		Santiago (RS)	29.18	54.85	41(5)
Argentina	ARGE	Santa Rosa	36.61	64.32	53(1)
		Ataliva Roca	37.05	64.28	54(2), 55(1)
		Entre Lihuel Calel	37.53	65.15	56(1)
		Winifreda	35.22	64.26	57(1), 58(1)

### Extraction of DNA, amplification and sequencing

Samples of *A*. *striatus* preserved in alcohol were used for extraction of total DNA from one individual of each colony, using the protocol of Fernandes-Salomão *et al*. (2005) [29]. The extracted DNA was used to amplify part of the mitochondrial *cytochrome oxidase subunit* I gene (COI) and part of the nuclear gene *wingless* (wg). The primer pair CO11-3F and CO12-4R [30] was used for amplification of the COI gene and the pair Wg578F [31] and Wg1032R [32] for the wg gene.

PCR was performed using 1U of *Taq* polymerase (Promega), dNTPs (0.25 mM each), MgCl2 (2.5 mM), 1X *Taq* buffer (Promega), primers (0.48 mM each) and 1 μL of DNA (50 ng) in a final volume of 25 μL. The amplification conditions included an initial denaturation step at 94°C for 3 min, followed by 35 cycles of 1 min at 94°C for DNA denaturation, 1 min at 53.5°C (COI) or 55°C (wg) for annealing of the primers and 72°C for 2 min (COI) or 1 min (wg) for primer extension followed by a final extension step at 72°C for 7 min.

The amplicons were sent to Macrogen Inc., South Korea (www.macrogen.com), purified and sequenced directly in both directions (forward and reverse) using the same primers as in the amplification reactions.

The forward and reverse strands were visually inspected and assembled using the program Consed [33]. Sequences were first translated into amino acid sequences to guarantee the homology of the sites and to exclude the possible presence of stop codons or indels. Thereafter the nucleotides were aligned using the ClustalW algorithm [34] in the MEGA5 program [35].

### Genetic diversity and structure of the population

The genetic diversity of each population sampled was investigated using the DNAsp 5.1 program [36] to assess nucleotide polymorphism, number of haplotypes (H), haplotype diversity (Hd), and nucleotide diversity (π).

The population structure was evaluated by spatial analysis of molecular variance (SAMOVA) implemented in the SAMOVA 1.0 program [37]. This analysis allows defining groups of populations without *a priori* information on population structure. The method is based on F-statistics and utilizes a simulated annealing procedure to define *K* population groups that are geographically homogeneous and genetically different from each other. Populations were split into two to twelve groups, and the apportionment of genetic variation and its cause was analyzed (F_CT_). The highest and statistically significant value of F_CT_ for a determined *K* was identified among the groups analyzed.

An analysis of molecular variance (AMOVA) was estimated by the genetic variation and fixation indices implemented in Arlequin 3.5 [38]. AMOVA was calculated with three hierarchical levels according to the population groups defined by SAMOVA. We also performed a second AMOVA considering sample sites, without any *a priori* population group, to test possible bias due our population grouping.

To assess whether genetic variation could be explained by isolation by distance, a correlation was performed between the logarithm of genetic distance and geographic distance by Mantel tests [39] using the program Alleles In Space (AIS) [40] with 10000 replications.

A gene genealogy was reconstructed in the program Network 4.6.1.1. (www.fluxus-engineering.com) using the median-joining algorithm to verify the existence of a relationship between the distribution of haplotypes and the geographic distribution of the analyzed samples of *A*. *striatus*.

### Historical demography and divergence

To determine if populations of *A*. *striatus* underwent recent population expansions or bottlenecks, we used Fu’s Fs [41] and Tajima’s D [42] neutrality tests in ARLEQUIN 3.5 for each population. The distribution of genetic differences between pairs of haplotypes (Mismatch distribution) was performed in the population that showed significant results for the neutrality tests considering the premise of panmictic populations. The observed distribution was compared with the one expected for the demographic expansion model and the spatial expansion model using the program ARLEQUIN 3.5. In general, multimodal distributions are consistent with demographic stability or multiple expansion events, while unimodal distributions commonly indicate that the population underwent a recent population and spatial expansion [38]. Fit of the models to our data was assessed by the significance of the sums of squared deviations (SSD) and Harpending’s raggedness index (Hri index).

To determine when major clades diverged and if the time of divergence corresponds to any of the known events in the Quaternary Period, we estimated the divergence times between clades following Seal et al. (2011) [43]. The time since the most recent common ancestor (TMRCA) was estimated by the Bayesian approach in MCMC chains using the program BEAST 1.6.1 [44]. The mutation rate of 1.455E-02 ± 1.25E-03 substitutions per site per million years, estimated specifically for the COI gene of ants [11], was used under an uncorrelated log-normal relaxed molecular clock. This approach was selected because an analysis in PAUP* [45] rejected the restricted or fixed molecular clock model (χ^2^ = 211.6; d.f. = 56; P < 0.001). Treating this as an intraspecific analysis, the identical sequences were removed and the coalescence of constant size model was used (coalescence: constant size). Analyzes were conducted using the SRD06 nucleotide substitution model [46], which allows that the third position of the codon has a substitution rate different from that estimated for the first and second positions of the codon. The analyses were performed by setting 30 million generations and 20% of the initial runs were excluded. A total of five independent analyses were conducted and subsequently combined and analyzed in the program TRACER v 1.5 [47], to verify the consistency and repeatability of the data by means of the effective sample size (ESS) >200.

### Modeling of the current and historical potential distribution

The potential current and quaternary distribution of *A*. *striatus* was determined using the maximum entropy algorithm in Maxent [48]. This model allows calculating the probability of occurrence of a species in a geographic area based on environmental variables for a given set of locations. To generate correlative models of the range of *A*. *striatus* under current and paleoclimates, we used 311 geo-referenced point locality data obtained from our own fieldwork (S1 Table). The climatic variables used to perform the modeling were: mean annual temperature (BIO1), temperature seasonality (standard deviation *100) (BIO4), mean temperature in the driest quarter (BIO9), annual precipitation (BIO12), precipitation seasonality (coefficient of variation *100) (BIO15), and precipitation in the warmest quarter (BIO18), available at the WorldClim database (http:\\www.worldclim.org) and with a spatial resolution of 2.5 arc-min [49]. These variables were selected based on the nesting biology, the thermoregulatory capacity of *A*. *striatus* and its symbiotic fungus [50], and to minimize the autocorrelation between the different WorldClim variables. The environmental dataset was clipped to the species’ approximate supposed distribution, following recommendations by Anderson and Raza [51]. Considering the spatial resolution used in the analysis, 57 of 311 points were independent. The algorithm removed duplicate points to perform the modeling. We modeled the distribution of *A*. *striatus* using 75% of the species data for training and 25% for testing the models, setting Maxent to run with random seed, a convergence threshold of 0.00001 with 500 interactions and 10,000 maximum number of background points. We selected the logistic output format to generate response curves jackknife results. In order to avoid spurious predictions, we defined a minimum training presence threshold, which is equal to the prediction of a model with a minimum value of presence for any occurrence. The modeling performance was evaluated using the average values of the 25% training gains through 10 bootstrap replicates and the mean of the area under the curve (AUC) of the receiver operating characteristic curve (ROC).

The model of potential current species distribution was trained and projected to three paleoclimate scenarios, one for Last Interglacial Period (LIG, 130 thousand years Before Present–B.P.) and two for the Last Glacial Maximum period (LGM – 21 thousand years B.P.), the MIROC (Model of Interdisciplinary Research on Climate) and CCSM3 (Community Climate System Model) from WorldClim database (http:\\www.worldclim.org) [49], in order to infer the appropriate predictive area for the occurrence of *A*. *striatus* during the Quaternary.

## Results

### Characterization and diversity of the sequences

A total of 922 unambiguous base pairs were sequenced from the mitochondrial COI gene in natural populations of *A*. *striatus*, and no stop codon or indel was observed. For the nuclear gene wingless, 338 base pairs were sequenced (KU162879-KU162939); however, this gene did not show any intraspecific polymorphism. Thus, all following analyses were only performed with the COI gene. All COI sequences were deposited in GenBank under the accession numbers KR605508–KR605635. Of the 66 variable sites in COI, 58 were informative for parsimony and eight were singletons. We identified 58 haplotypes. The difference in the number of nucleotides between sequences averaged 12.96 nucleotides (min—max = 0–37), and the maximum divergence between two sequences was 4.01%. Although, the definition of what is a natural population is always an arbitrary issue. The population evaluations that follow inhere should be viewed with thoughtfulness since they could bear possible bias introduced by our population grouping strategy. Haplotype diversity (Hd) was 0.908 ± 0.021 and total nucleotide diversity (π) was 0.01406 ± 0.00094 across all populations. The populations presenting the highest and lowest haplotype diversity were ARGE and FLOR, respectively. The population FLOR also showed the lowest nucleotide diversity, while the highest was observed in NORT (Table 2).

**Table 2:** Genetic diversity and neutrality tests for each population of *Acromyrmex striatus*. Significant values are presented in bold.

Population	N	H	Hd	π	Neutrality tests			
					Tajima’s D	*p*-value	Fu’s Fs	*p*-value
FLOR	09	01	0.000 ± 0.000	0.00000 ± 0.000	0.00000	1.00000	0.00000	n.a
CONT	06	02	0.533 ± 0.172	0.00058 ± 0.000	0.85057	0.88170	0.62543	0.37000
CABO	10	03	0.511 ± 0.164	0.00137 ± 0.000	-0.39990	0.36850	0.94880	0.69820
LAGU	10	05	0.667 ± 0.163	0.00207 ± 0.001	-1.39868	0.09030	-0.70594	0.25180
SOUT	09	05	0.722 ± 0.159	0.00193 ± 0.001	**-1.79752**	**0.01130**	-1.11299	0.13920
EXTR	08	06	0.607 ± 0.164	0.00221 ± 0.001	-0.56068	0.31290	1.61176	0.81340
NORT	09	07	0.944 ± 0.070	0.01344 ± 0.004	1.72582	0.98130	0.72790	0.60250
MOTD	12	08	0.894 ± 0.078	0.00251 ± 0.002	**-1.75016**	**0.02680**	**-3.45722**	**0.00970**
RIOG	14	10	0.956 ± 0.038	0.00365 ± 0.002	-0.95645	0.17710	-4.07787	0.01190
INTE	12	08	0.894 ± 0.078	0.00526 ± 0.002	-0.81319	0.22000	-1.13556	0.25360
SAMA	09	06	0.833 ± 0.127	0.00368 ± 0.001	0.10765	0.56430	-0.87706	0.24990
WEST	13	04	0.423 ± 0.164	0.00139 ± 0.001	-1.24415	0.11130	0.02744	0.47760
ARGE	07	06	0.952 ± 0.096	0.00424 ± 0.002	0.33464	0.64290	-1.64228	0.10910

N: number of samples; H: number of haplotypes; Hd: haplotype diversity; π: nucleotide diversity; ±: standard deviation.

### Phylogenetic relationships and geographic structure

**Fig 2:**
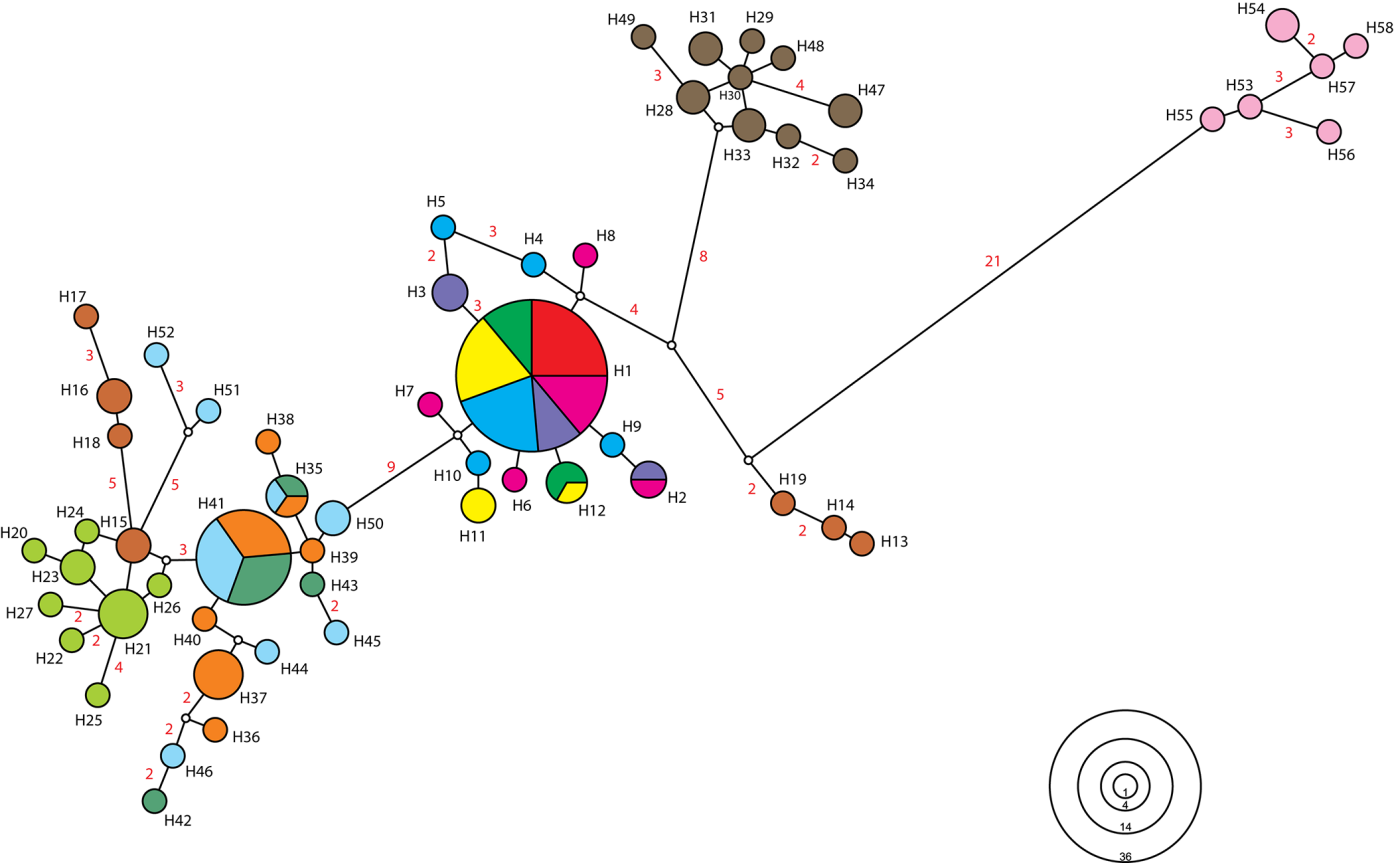
Genealogical relationship inferred for 58 haplotypes of the mitochondrial COI gene of *Acromyrmex striatus* estimated by the median-joining algorithm. The areas are proportional to the frequency of haplotypes, which ranged from 1 to 36. Colors represent each population sampled, and correspond with the colors of Fig 1. The numbers of haplotypes of *A*. *striatus* correspond with those shown in Table 1. Numbers in red indicate mutational steps between haplotypes greater than one. Five haplogroups can be viewed. The small white circles represent lost or unsampled haplotypes.

Fig 2 shows the phylogenetic relationships among haplotypes of *A*. *striatus* with five geographically structured haplogroups. The populations of *A*. *striatus* collected in the coastal region of Santa Catarina were all included in the same haplogroup, including 12 haplotypes, with haplotype H1 (n = 36) being the most abundant (Fig 2). Similarly, samples collected in Argentina formed a single haplogroup with high haplotype diversity. Samples from the southern coastline of Rio Grande do Sul (RIOG) form a haplogroup separate from samples collected in the interior and on the coast of Rio Grande do Sul. Three exclusive haplotypes (H13, H14 and H19) were identified in samples from the NORT population (both locations are situated along the northern coast of the state of Rio Grande do Sul) and form a haplogroup isolated from the other populations. The remaining populations of the interior and the coast of Rio Grande do Sul formed a distinct haplogroup, with the most abundant haplotype being H41 (n = 14). Haplotype distribution shows high regional structuring of *A*. *striatus* populations (S1 Fig), where haplogroups are connected by nine or more mutational steps.

**Fig 3:**
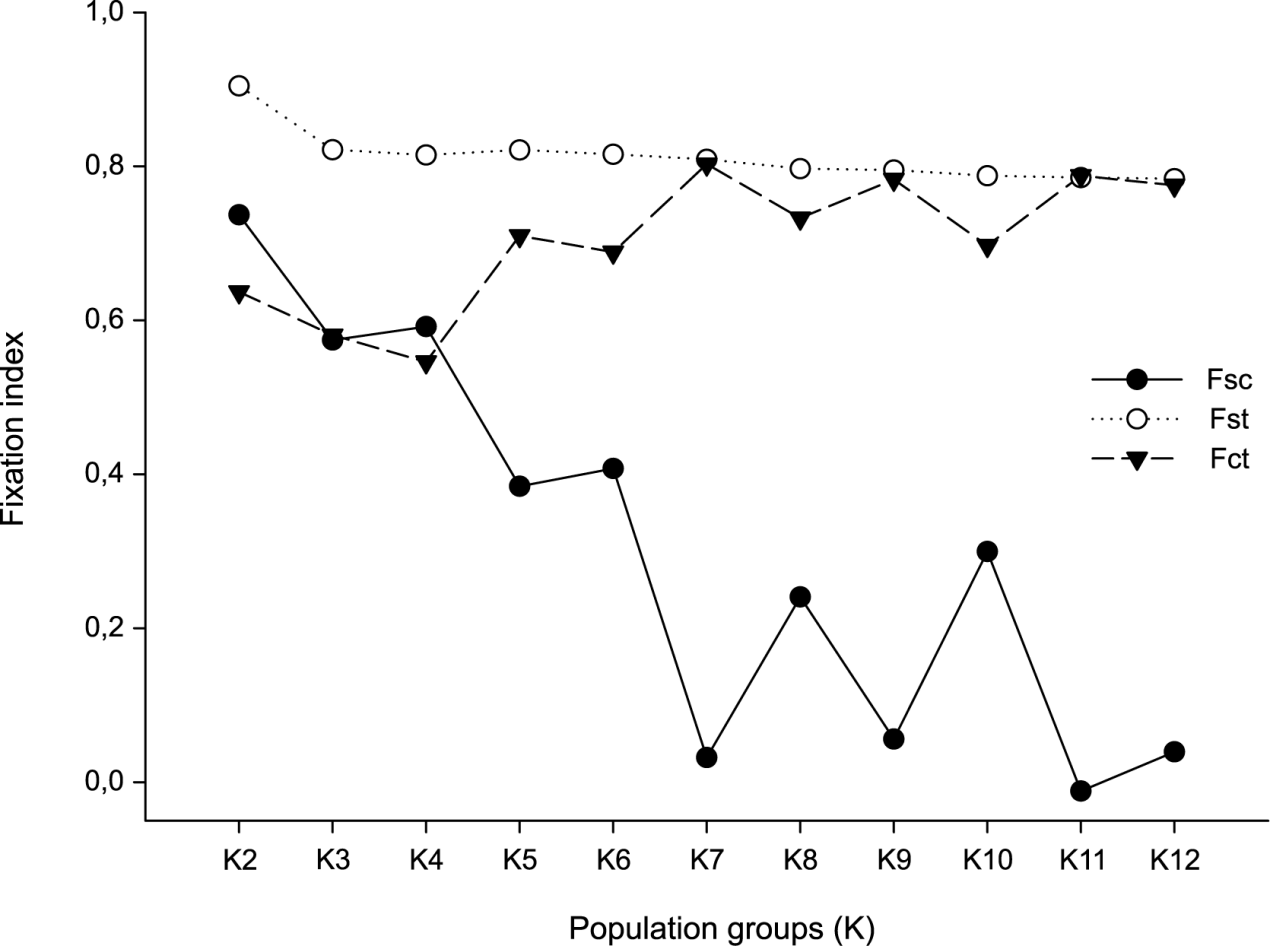
Spatial analysis of molecular variance (SAMOVA) of the 13 populations of *Acromyrmex striatus*. The fixation indexes were calculated independently for each group *K*. The most significant value of the fixation index F_CT_ for a given *K* reports the best grouping of populations of *A*. *striatus*.

SAMOVA allowed identifying the maximum genetic differentiation between the groups and therefore estimating potential barriers to gene flow. The split in *K* = 7 groups showed the highest value of F_CT_ (Fig 3) and the subdivision scheme: [FLOR, CABO, LAGU, SOUT, EXTR] [CONT] [NORT] [MOTD] [RIOG] [INTE, SAMA, WEST] [ARGE]. Based on the groups suggested by SAMOVA, the AMOVA showed that most of the genetic variation was attributed to differences among population groups or between sampled regions (Table 3). The AMOVA results considering sampling sites without the population grouping also showed that most of the genetic variation was attributed to differences among population groups (S2 Table). The population groups identified by SAMOVA (K = 7) match the geographical location of populations and adjacent populations were grouped together. Pairwise F_ST_ values varied between 0.9494 and 0.0000 and were mostly significant (*p* < 0.05). In general, the lowest values of F_ST_ were observed between neighboring populations. F_ST_ values between populations of Santa Catarina were not significant, suggesting that there is no significant lack of gene flow between these populations (S3 Table).

**Table 3:** Analysis of molecular variance (AMOVA) for the species *Acromyrmex striatus*, performed with three hierarchical levels, considering the groups formed by SAMOVA.

Source of variation	df	Percentage of variation
Among groups	6	80.28
Among populations within groups	6	0.63
Within groups	115	19.09

df: degrees of freedom. All values were significant at *p* < 0.01.

The logarithm of genetic distance and the geographical distance were significantly correlated (Mantel test, r = 0.5662; *p* < 0.01), indicating that the genetic differentiation between *A*. *striatus* populations may be partially attributed to isolation by distance. An analysis after exclusion of the most distant population from Argentina still yielded a significant result (r = 0.5031; *p* <0.01).

### Historical demography and molecular dating

Neutrality tests were conducted for each of the 13 populations of *A*. *striatus* and the results are shown in the Table 2. Among the 13 populations analyzed, only one (MOTD population) showed significant, negative values for both Fu’s Fs (*p* < 0.01) and Tajima’s D (*p* < 0.05) tests. These results indicate that haplotype frequencies for MOTD differ from those expected by the neutral evolution hypothesis and suggest a recent population expansion. The unimodal distribution of pairwise haplotype differences in the mismatch distribution analysis of MOTD gave a similar result (S2 Fig), and the observed distribution pattern fits well with the spatial expansion (SDD = 0.00758; *p* = 0.70; *Hri* = 0.06084; *p* = 0.62) and the demographic expansion models (SDD = 0.00758; *p* = 0.70; *Hri* = 0.06084; *p* = 0.66).

Table 4 presents the time of divergence between estimated clades by means of the Bayesian approach. Time to the most recent common ancestor of populations from Brazil and Argentina was ~2.5 million years. Divergence times for all Brazilian populations was ~1.531 million years and for populations from the interior of Rio Grande do Sul (including MOTD and part of NORT) ~664,000 years. Populations from the coast of Santa Catarina and the extreme south of Rio Grande do Sul (RIOG) are relatively younger, with divergence times of 336,100 and 324,500 years, respectively (Table 4).

**Table 4:** Estimates of the divergence time of the most recent common ancestor between the haplogroups of *Acromyrmex striatus* (in million years). Estimates were based on the mutation rate for the mitochondrial COI gene in ants (0.01455 bp per million years).

Haplogroup	TMRCA	95% CI	ESS
Argentina	0.3016	(0.085–0.593)	23850
Brazil	1.5310	(0.794–2.583)	5769
Cassino	0.3245	(0.011–0.615)	18845
SC Coast	0.3361	(0.127–0.611)	18370
Rio Grande do Sul State	0.6644	(0.345–1.054)	25659
Torres	0.1578	(0.017–0.368)	26175
All	2.5913	(1.189–4.704)	16432

TMRCA: Time to most recent common ancestor; CI: Confidence interval; ESS: Effective sample size.

### Current and historical potential distribution

**Fig 4:**
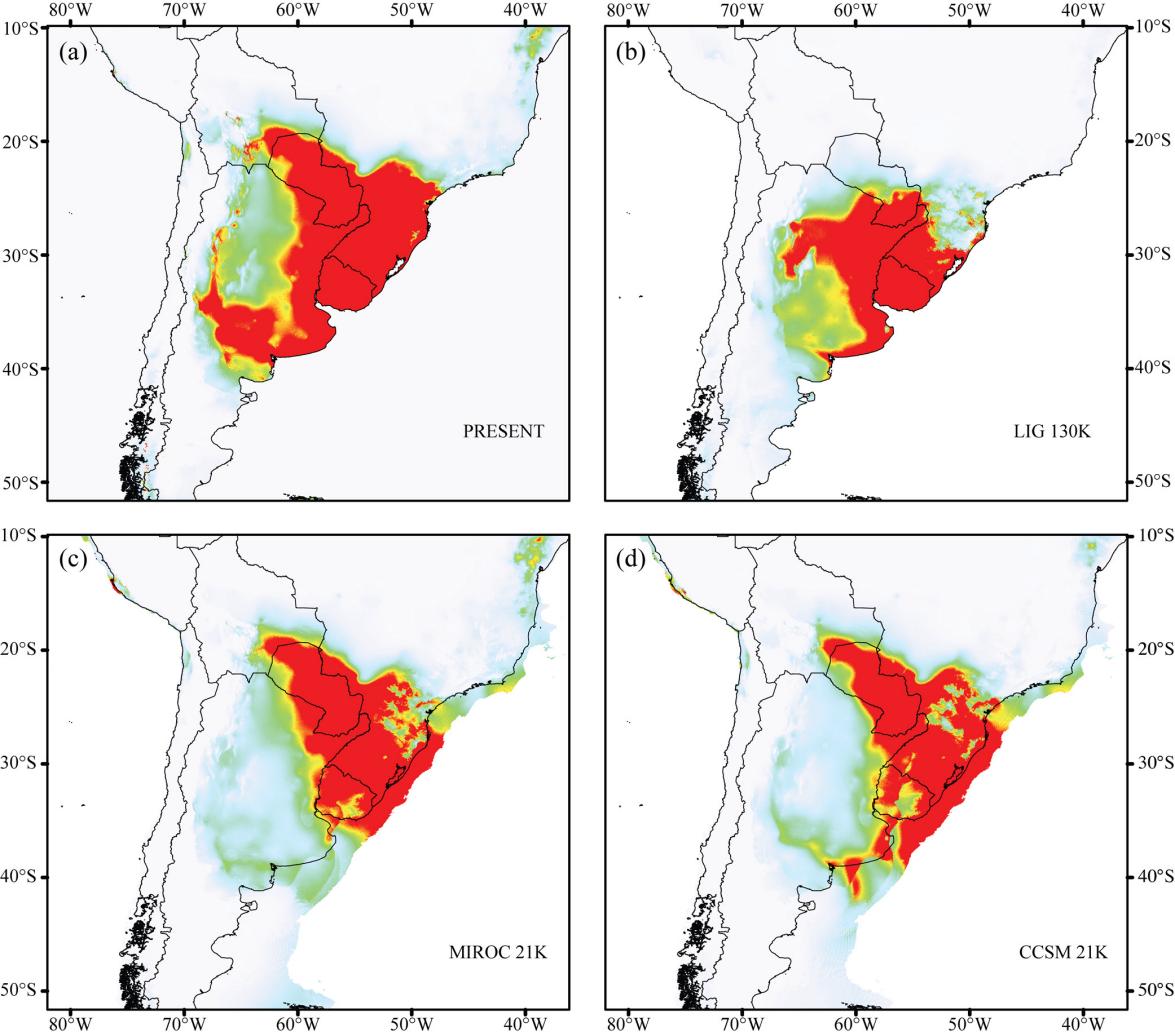
Geographical distribution for climatically predicted areas for the occurrence of *Acromyrmex striatus* based on current and past bioclimatic variables. (a) potential distribution under current conditions, (b) LIG 130K: last interglacial period (130,000 years before present), (c) MIROC 21k and (d) CCSM 21k: last glacial maximum (21,000 years before present). Warm colors (red/yellow) represent areas with high probability of *A*. *striatus*.

The modeled current potential distribution (Fig 4) widely overlapped with the current known distribution of *A*. *striatus*, although it included a few areas that are not suitable for this ant (e.g., Atlantic Forest areas north of the state of Santa Catarina). The models achieved over 2.5 regularized gain values indicating good fit to presence data. The AUC was high, with a mean value of 0.981 and a standard deviation of 0.009, showing no over-fitting around the presence data. Projection of the model for the potential occurrence of *A*. *striatus* on the paleoclimatic parameters of the Last Glacial Maximum (LGM) and Last Interglacial Period (LIG) recovered appropriate potential areas for the occurrence of *A*. *striatus* (Fig 4). Paleodistribution models suggest that the potential habitat of *A*. *striatus* may have decreased during the LIG and expanded during the LGM. Estimated areas with a high probability of occurrence during the LGM even surpassed the present coastline, which can be explained by the lower sea levels during the LGM, which increased the Atlantic coastline by more than 120 km (Fig 4).

## Discussion

The results obtained in the present study clarify the evolutionary history and the pattern of genetic variability of *A*. *striatus*, a species associated with vegetation of the open plains. The observed phylogeographic pattern appears to reflect both ecophysiological traits of the studied species and isolation by distance.

Across its range, populations are genetically highly structured, presumably reflecting long-lasting isolation of population groups in the past. Populations within the same geographical region are genetically similar, suggesting restricted gene flow between geographically separated groups of populations. AMOVA confirmed the existence of five previously defined population groups (see Table 3), and SAMOVA identified seven potential barriers to gene flow that may have shaped the genetic differentiation among regions in the past (see Results section). One of the detected barriers separates the coastal populations from inland populations, while gene flow appears unrestricted among coastal populations of Santa Catarina state, at the northern edge of *A*. *striatus* distribution. Populations on Santa Catarina’s island (FLOR) shared haplotypes with coastal populations, matching the observation that a few kilometers of sea do not represent an impassable barrier for flying insects [30, 43].

A similar pattern of unrestricted gene flow among coastal populations was also observed in the endemic sand dune ant *Mycetophylax simplex* [52], which occurs in sympatry with *A*. *striatus* in parts of its coastal range. This congruency may suggest that open sandy habitats, free of obvious geographical barriers, facilitate ant dispersion over long distances. However, *A*. *striatus* populations in the southern part of the coastal plain were genetically structured in contrast to those of *M*. *simplex*. This discordance might be due to differences between these two species in dispersal capabilities and habit requirements. *M*. *simplex* is thought to disperse over several kilometers whereas *A*. *striatus* was suggested to disperse only over short distances [52]. Furthermore, *M*. *simplex* nests always in open areas, which are abundant on the coast. In contrast, *A*. *striatus* nests on open, well-drained sites close to shrubby vegetation, which are patchily distributed on the coast.

Our study did not identify explicit physical barriers to gene flow. Although able to fly, queens of *A*. *striatus* have low dispersion capability [53] and successfully establish nests only in areas with sandy, well-drained soil, with little organic matter [54]. Selection of suitable soil for colony establishment is highly relevant for leaf-cutting ants [50]. Thus, the observed phylogeographic pattern might reflect the historical geomorphology of the two plains (coastal and inland plains) where *A*. *striatus* occurs. Both plains are characterized by sandy, permeable soils, and are influenced by strong winds. The coastal plains are geologically more recent and formed mainly of sandy soils. The Pampas are older environments with several different types of soil [23, 55] and sandy soils are patchily distributed. This heterogeneity of the landscape and the distribution of the preferred nesting habitat may well explain the structured phylogeographical pattern of *A*. *striatus* and matches similar findings in subterranean rodents of the genus *Ctenomys* [20, 21], and *Petunia* [56], and *Calibrachoa* plants from the same habitat [23].

Some of distance measures and F_ST_ values, mainly between Argentine populations, were quite extreme (>4% of sequence divergence and > 0.9 pairwise F_ST_, see S3 Table). Such results can be an indicative of possible presence of cryptic species, but further analysis would be needed. The combination of high haplotype and nucleotide diversity in *A*. *striatus* is related to historically large and stable populations [57]. In fact, neutrality tests did not identify deviations of the neutral evolution hypothesis for 12 from 13 studied populations. This indicates that *A*. *striatus* did not experience periods of rapid population expansion or bottlenecks (except for the MOTD population, see results), but that the species remained demographically stable. Thus, our study suggests that the colonization of open vegetation in the plains by *A*. *striatus* occurred gradually and was influenced by factors such as climatic events at the end of the Pliocene and throughout the Quaternary Period.

Reconstruction of the potential distribution area of *A*. *striatus* suggests that suitable habitats underwent a moderate expansion and contraction during the glacial periods (Fig 4 –LGM, 21,000 years B.P.). This reaffirms the hypothesis that open vegetation zones expanded to the north of South America [14]. Paleodistribution models indicate a decrease of the suitable areas for *A*. *striatus* during the interglacial period (Fig 4B—LIG, 130,000 years B.P.), possibly associated with increased humidity and expansion of forest environments [15]. However, the area of potential occurrence of *A*. *striatus* during the glacial and interglacial periods was quite similar to the current estimated suitable areas. The current populations of *A*. *striatus* may be derived from a large population, which remained demographically stable over evolutionary time. Possible population expansions and reductions in the past may not have eliminated the genetic imprint of demographic stability for *A*. *striatus* populations. The historical demographic stability observed in the present study, as well as the phylogenetic position of *A*. *striatus* as a sister group of the other leaf-cutting ants [28], agree with the hypothesis by Fowler (1983) that leaf-cutter ants must have originated and diversified in open environments in southern South America [58].

It has been thought that events during the Pleistocene have acted as main trigger of species diversification in the Neotropics [59]. However, phylogeographic studies on Atlantic Forest species based on molecular clock have suggested that species diversification and dispersion may have occurred already before the Pleistocene [11–13]. Our results confirm this observation, since the estimated age for the TMRCA of *A*. *striatus* haplotypes falls into the Pliocene. The age of major clades of *A*. *striatus* seems to match the estimated time of the formation of coastal plains in Rio Grande do Sul by gradual depositions of sand barriers along the coast during the Pleistocene and Holocene around 400,000 B.P. [60]. According to the haplotype frequencies, the central and northern coastal populations (NORT; MOTD) are separated from those in the south (RIOG). This suggests that the RIOG population is derived from a lineage independent of other lineages present in Rio Grande do Sul (north coast and inland populations). The time of divergence of ~324.500 years corresponds to the age of formation of the sandy barrier where this population occurs today (see [60]). The remaining costal populations occupy sand ridges formed during secondary marine transgressive-regressive events and are therefore younger.

In conclusion, our results suggest that populations of *A*. *striatus* are geographically structured as a result of stable historical demography during the evolutionary time and low dispersion capacity across sandy soil patches. Isolation by distance appears to have a strong influence and suggests equilibrium between migration and genetic drift in this ant species. These results are consistent with the hypothesis that the Pampas and the coastal region of southern Brazil were not strongly affected by the expansion of forests during the interglacial periods. Due to the diversity of responses observed in species inhabiting open vegetation environments, more species from such environments need to be studied to better understand the general processes that governed the diversification of these ecosystems.

## Supporting Information

S1 Fig Geographical distribution and genealogical relationship of *Acromyrmex striatus*. The haplotype network (Fig 2) of the mitochondrial COI gene of *A*. *striatus* is superimposed on the map (Fig 1) approximately on the sampling points.
(TIF)

S2 Fig Mismatch distributions for the population MOTD of *Acromyrmex striatus*. Histograms represent the observed frequencies of the pair-to-pair differences between haplotypes and lines represent the simulated curve for the population MOTD under the demographic expansion (solid line) and spatial expansion models (dashed line).
(TIF)

S1 Table List of 311 new records of nests of *A*. *striatus* collected by us in Brazil and Argentina between February 2009 and August 2011. (PDF)

S2 Table Analysis of molecular variance (AMOVA) for the *A*. *striatus*, performed with three hierarchical levels, considering each 38 sites in Brazil e Argentina (populations), considering the groups formed by SAMOVA (7 groups). (PDF)

S3 Table F_ST_ values of pair-wise comparisons between populations of *Acromyrmex striatus*, estimated from the mitochondrial COI gene. Values shown in bold are significant (p < 0.05).
(PDF)
