# Supplementary figures and images for: Integrating Paleodistribution Models and Phylogeography in the Grass-Cutting Ant Acromyrmex striatus (Hymenoptera: Formicidae) in Southern Lowlands of South America
Source: PLoS One. 2016 Jan 6;11(1):e0146734. doi: 10.1371/journal.pone.0146734 (PMC4703384; doi:10.1371/journal.pone.0146734)

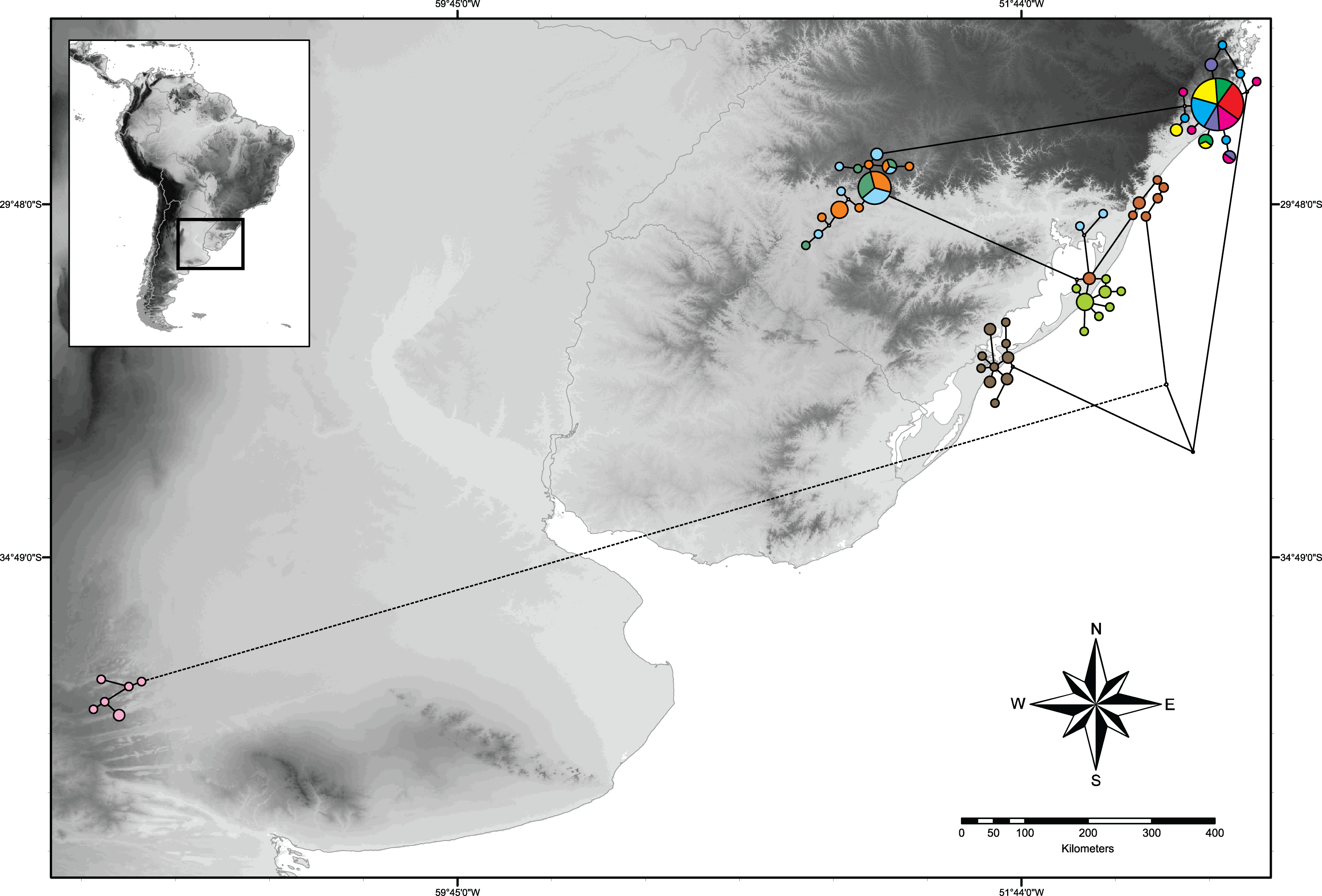

Supplement: S1 Fig — The haplotype network (Fig 2) of the mitochondrial COI gene of A. striatus is superimposed on the map (Fig 1) approximately on the sampling points. (TIF) [file pone.0146734.s001.tif]

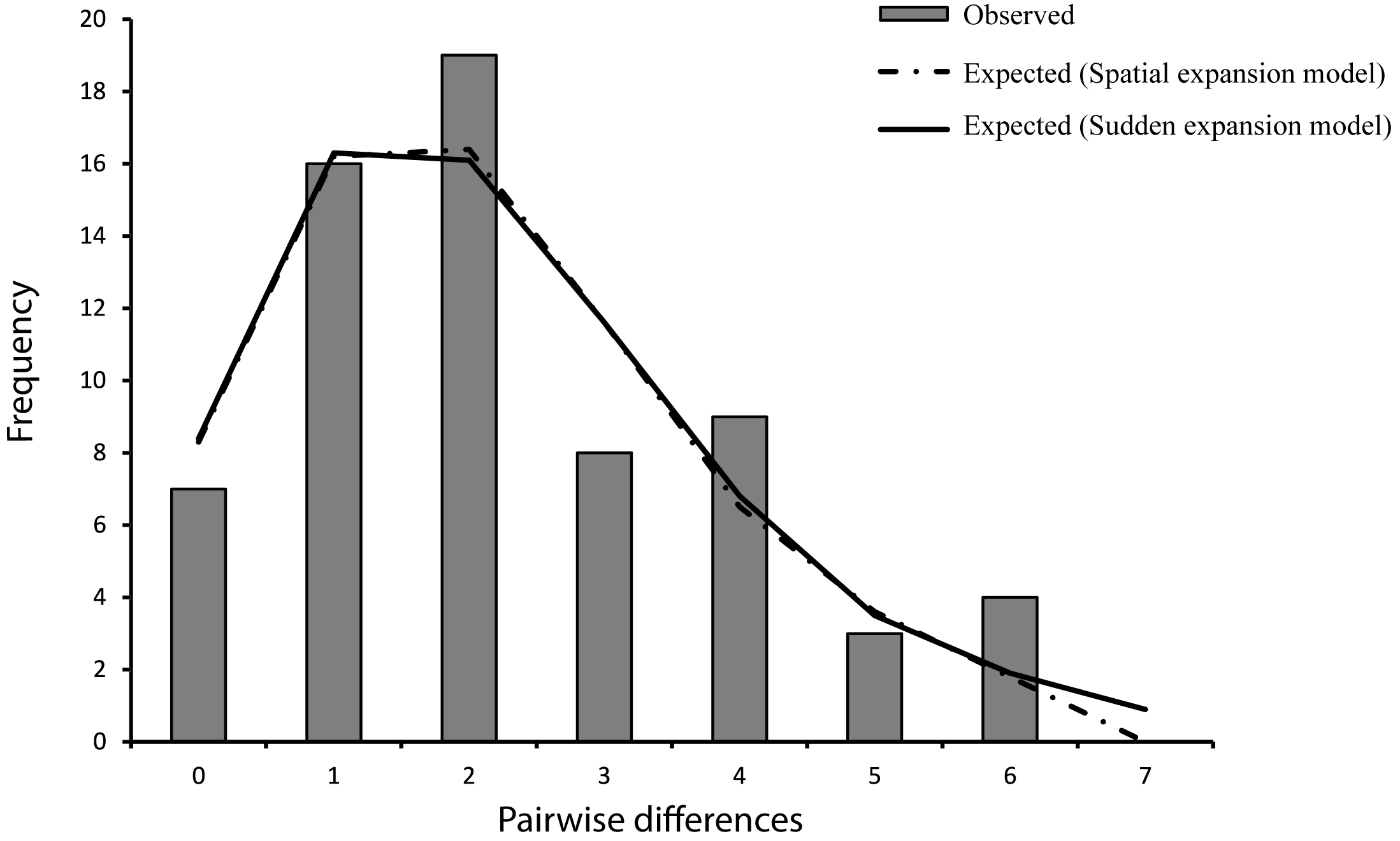

Supplement: S2 Fig — Histograms represent the observed frequencies of the pair-to-pair differences between haplotypes and lines represent the simulated curve for the population MOTD under the demographic expansion (solid line) and spatial expansion models (dashed line). (TIF) [file pone.0146734.s002.tif]
