# Supplementary material for: Integrating Paleodistribution Models and Phylogeography in the Grass-Cutting Ant Acromyrmex striatus (Hymenoptera: Formicidae) in Southern Lowlands of South America
Source: PLoS One. 2016 Jan 6;11(1):e0146734. doi: 10.1371/journal.pone.0146734 (PMC4703384; doi:10.1371/journal.pone.0146734)
Supplement: S1 Table — (PDF) [file pone.0146734.s003.pdf]

Table S1 - List of 311 new records of nests of *A. striatus* collected by us in Brazil and Argentina between February 2009 and August 2011.

Locality	Geographical coodinate	
Araranguá - SC	S28° 57’ 11.3”	W49° 22’ 29.6”
Araranguá - SC	S28° 56’ 36.4”	W49° 27’ 59.4”
Araranguá - SC	S28° 57’ 14.4”	W49° 22’ 33.0”
Araranguá - SC	S28° 57’ 15.1”	W49° 22’ 33.4”
Araranguá - SC	S28° 57’ 16.1”	W49° 22’ 34.3”
Araranguá - SC	S28° 57’ 18.1”	W49° 22’ 35.4”
Araranguá - SC	S28° 57’ 21.1”	W49° 22’ 38.6”
Araranguá - SC	S28° 57’ 07.7”	W49° 22’ 26.8”
Araranguá - SC	S28° 57’ 06.5”	W49° 22’ 26.4”
Araranguá - SC	S28° 57’ 06.9”	W49° 22’ 26.0”
Araranguá - SC	S28° 57’ 06.7”	W49° 22’ 28.3”
Araranguá - SC	S28° 56’ 12.3”	W49° 21’ 25.9”
Araranguá - SC	S28° 56’ 12.1”	W49° 21’ 23.5”
Araranguá - SC	S28° 56’ 10.5”	W49° 21’ 20.5”
Araranguá - SC	S28° 56’ 13.2”	W49° 21’ 21.2”
Araranguá - SC	S28° 56’ 03.9”	W49° 21’ 13.6”
Araranguá - SC	S28° 56’ 02.6”	W49° 21’ 12.5”
Araranguá - SC	S28° 56’ 02.0”	W49° 21’ 08.8”
Araranguá - SC	S28° 55’ 59.1”	W49° 21’ 06.3”
Arroio do Silva - SC	S29° 02’ 53.2”	W49° 28’ 21.2”
Arroio do Silva - SC	S29° 02’ 52.0”	W49° 28’ 20.1”
Arroio do Silva - SC	S29° 02’ 50.1”	W49° 28’ 17.5”
Arroio do Silva - SC	S29° 02’ 47.5”	W49° 28’ 16.6”
Arroio do Silva - SC	S29° 02’ 43.1”	W49° 28’ 11.7”
Arroio do Silva - SC	S29° 02’ 41.8”	W49° 28’ 10.8”
Arroio do Silva - SC	S29° 02’ 39.7”	W49° 28’ 09.0”
Arroio do Silva - SC	S29° 02’ 38.0”	W49° 28’ 09.6”
Arroio do Silva - SC	S29° 02’ 38.8”	W49° 28’ 07.5”
Arroio do Silva - SC	S29° 02’ 36.8”	W49° 28’ 09.0”
Arroio do Silva - SC	S29° 02’ 36.1”	W49° 28’ 05.8”
Arroio do Silva - SC	S29° 00’ 47.0”	W49° 26’ 21.4”
Arroio do Silva - SC	S29° 00’ 47.6”	W49° 26’ 22.3”
Arroio do Silva - SC	S29° 00’ 48.8”	W49° 26’ 20.0”
Arroio do Silva - SC	S29° 00’ 50.0”	W49° 26’ 20.5”
Arroio do Silva - SC	S29° 00’ 50.1”	W49° 26’ 22.3”
Arroio do Silva - SC	S29° 00’ 50.9”	W49° 26’ 22.5”
Arroio do Silva - SC	S29° 00’ 48.5”	W49° 26’ 24.3”
Arroio do Silva - SC	S29° 00’ 49.9”	W49° 26’ 26.0”
Arroio do Silva - SC	S29° 00’ 54.1”	W49° 26’ 24.5”
Arroio do Silva - SC	S29° 00’ 59.2”	W49° 26’ 27.6”
Arroio do Silva - SC	S29° 00’ 59.3”	W49° 26’ 30.6”

Gaivota - SC	S29^{o} 11’ 03.5”	W49^{o} 35’ 57.7”
Gaivota - SC	S29^{o} 11’ 05.4”	W49^{o} 35’ 58.0”
Gaivota - SC	S29^{o} 11’ 05.5”	W49^{o} 35’ 58.3”
Gaivota - SC	S29^{o} 11’ 05.9”	W49^{o} 35’ 58.5”
Gaivota - SC	S29^{o} 11’ 05.4”	W49^{o} 35’ 59.4”
Gaivota - SC	S29^{o} 11’ 06.6”	W49^{o} 35’ 58.6”
Gaivota - SC	S29^{o} 11’ 07.4”	W49^{o} 35’ 59.4”
Gaivota - SC	S29^{o} 11’ 08.9”	W49^{o} 36’ 00.9”
Gaivota - SC	S29^{o} 11’ 10.6”	W49^{o} 36’ 02.0”
Gaivota - SC	S29^{o} 11’ 08.3”	W49^{o} 36’ 02.3”
Gaivota - SC	S29^{o} 11’ 00.7”	W49^{o} 35’ 54.6”
Laguna - SC	S28^{o} 36’ 30.4”	W48^{o} 50’ 09.4”
Laguna - SC	S28^{o} 36’ 30.5”	W48^{o} 50’ 07.7”
Laguna - SC	S28^{o} 36’ 28.5”	W48^{o} 50’ 07.1”
Laguna - SC	S28^{o} 36’ 28.8”	W48^{o} 50’ 06.8”
Laguna - SC	S28^{o} 36’ 28.2”	W48^{o} 50’ 05.8”
Laguna - SC	S28^{o} 36’ 27.4”	W48^{o} 50’ 04.2”
Laguna - SC	S28^{o} 36’ 27.2”	W48^{o} 50’ 03.2”
Laguna - SC	S28^{o} 36’ 26.1”	W48^{o} 50’ 02.4”
Laguna - SC	S28^{o} 36’ 25.5”	W48^{o} 50’ 03.0”
Laguna - SC	S28^{o} 36’ 24.4”	W48^{o} 50’ 00.3”
Ilhas - SC	S28^{o} 54’ 40.8”	W49^{o} 21’ 36.8”
Ilhas - SC	S28^{o} 54’ 38.3”	W49^{o} 21’ 50.8”
Ilhas - SC	S28^{o} 54’ 38.4”	W49^{o} 21’ 51.3”
Ilhas - SC	S28^{o} 54’ 12.2”	W49^{o} 19’ 13.9”
Ilhas - SC	S28^{o} 54’ 11.2”	W49^{o} 19’ 13.3”
Ilhas - SC	S28^{o} 54’ 09.4”	W49^{o} 19’ 10.0”
Ilhas - SC	S28^{o} 54’ 14.5”	W49^{o} 19’ 20.2”
Ilhas - SC	S28^{o} 54’ 14.2”	W49^{o} 19’ 19.2”
Ilhas - SC	S28^{o} 54’ 13.6”	W49^{o} 19’ 17.6”
Ilhas - SC	S28^{o} 54’ 12.1”	W49^{o} 19’ 14.0”
Ilhas - SC	S28^{o} 54’ 10.7”	W49^{o} 19’ 12.9”
Ilhas - SC	S28^{o} 54’ 09.5”	W49^{o} 19’ 10.6”
Ilhas - SC	S28^{o} 54’ 08.1”	W49^{o} 19’ 09.4”
Rincão - SC	S28^{o} 48’ 55.9”	W49^{o} 12’ 24.3”
Rincão - SC	S28^{o} 48’ 54.8”	W49^{o} 12’ 22.7”
Rincão - SC	S28^{o} 48’ 53.3”	W49^{o} 12’ 17.4”
Rincão - SC	S28^{o} 48’ 50.0”	W49^{o} 12’ 15.1”
Rincão - SC	S28^{o} 48’ 47.4”	W49^{o} 12’ 13.0”
Rincão - SC	S28^{o} 48’ 47.0”	W49^{o} 12’ 09.0”
Rincão - SC	S28^{o} 48’ 45.4”	W49^{o} 12’ 06.1”
Rincão - SC	S28^{o} 48’ 43.1”	W49^{o} 12’ 05.3”
Rincão - SC	S28^{o} 48’ 41.4”	W49^{o} 12’ 04.5”
Rincão - SC	S28^{o} 48’ 38.0”	W49^{o} 12’ 00.9”
Itapirubá - SC	S28^{o} 19’ 10.4”	W48^{o} 42’ 31.4”
Itapirubá - SC	S28^{o} 19’ 02.6”	W48^{o} 42’ 29.7”

Itapirubá - SC	S28^{o} 18’ 56.5”	W48^{o} 42’ 28.1”
Itapirubá - SC	S28^{o} 18’ 52.9”	W48^{o} 42’ 25.6”
Itapirubá - SC	S28^{o} 18’ 47.9”	W48^{o} 42’ 23.9”
Itapirubá - SC	S28^{o} 18’ 46.0”	W48^{o} 42’ 23.3”
Itapirubá - SC	S28^{o} 18’ 42.5”	W48^{o} 42’ 22.3”
Itapirubá - SC	S28^{o} 18’ 39.3”	W48^{o} 42’ 20.4”
Itapirubá - SC	S28^{o} 18’ 36.2”	W48^{o} 42’ 20.2”
Itapirubá - SC	S28^{o} 18’ 30.4”	W48^{o} 42’ 18.3”
Garopaba - SC	S27^{o} 59’ 42.4”	W48^{o} 37’ 55.5”
Garopaba - SC	S27^{o} 59’ 41.3”	W48^{o} 37’ 55.1”
Garopaba - SC	S27^{o} 59’ 40.0”	W48^{o} 37’ 55.3”
Garopaba - SC	S27^{o} 59’ 36.9”	W48^{o} 37’ 54.7”
Garopaba - SC	S27^{o} 59’ 34.3”	W48^{o} 37’ 54.6”
Garopaba - SC	S27^{o} 59’ 29.1”	W48^{o} 37’ 53.8”
Garopaba - SC	S27^{o} 59’ 15.0”	W48^{o} 37’ 53.5”
Garopaba - SC	S27^{o} 59’ 18.6”	W48^{o} 37’ 54.8”
Garopaba - SC	S27^{o} 59’ 13.7”	W48^{o} 37’ 49.8”
Garopaba - SC	S27^{o} 59’ 08.5”	W48^{o} 37’ 48.6”
Ibiraquera - SC	S28^{o} 09’ 47.3”	W48^{o} 39’ 22.7”
Ibiraquera - SC	S28^{o} 09’ 48.0”	W48^{o} 39’ 23.1”
Ibiraquera - SC	S28^{o} 09’ 50.0”	W48^{o} 39’ 24.5”
Ibiraquera - SC	S28^{o} 09’ 50.9”	W48^{o} 39’ 25.3”
Ibiraquera - SC	S28^{o} 09’ 53.6”	W48^{o} 39’ 25.7”
Ibiraquera - SC	S28^{o} 09’ 55.1”	W48^{o} 39’ 26.7”
Ibiraquera - SC	S28^{o} 09’ 57.0”	W48^{o} 39’ 27.6”
Ibiraquera - SC	S28^{o} 09’ 59.6”	W48^{o} 39’ 29.0”
Ibiraquera - SC	S28^{o} 10’ 00.5”	W48^{o} 39’ 29.1”
Ibiraquera - SC	S28^{o} 10’ 02.4”	W48^{o} 39’ 30.2”
Pinheira - SC	S27^{o} 52’ 31.3”	W48^{o} 36’ 02.4”
Pinheira - SC	S27^{o} 52’ 27.2”	W48^{o} 36’ 03.4”
Pinheira - SC	S27^{o} 52’ 24.1”	W48^{o} 36’ 05.4”
Pinheira - SC	S27^{o} 52’ 21.0”	W48^{o} 36’ 07.6”
Pinheira - SC	S27^{o} 52’ 15.5”	W48^{o} 36’ 08.6”
Sonho - SC	S27^{o} 50’ 32.1”	W48^{o} 35’ 14.8”
Sonho - SC	S27^{o} 50’ 25.6”	W48^{o} 35’ 15.3”
Sonho - SC	S27^{o} 50’ 24.0”	W48^{o} 35’ 15.6”
Sonho - SC	S27^{o} 50’ 21.7”	W48^{o} 35’ 14.7”
Sonho - SC	S27^{o} 50’ 19.9”	W48^{o} 35’ 15.8”
Moçambique - SC	S27^{o} 29’ 14.4”	W48^{o} 23’ 28.8”
Moçambique - SC	S27^{o} 29’ 10.9”	W48^{o} 23’ 25.2”
Moçambique - SC	S27^{o} 29’ 09.7”	W48^{o} 23’ 22.6”
Moçambique - SC	S27^{o} 29’ 02.0”	W48^{o} 23’ 10.3”
Moçambique - SC	S27^{o} 28’ 55.6”	W48^{o} 23’ 08.5”
Moçambique - SC	S27^{o} 28’ 55.7”	W48^{o} 23’ 14.7”
Joaquina - SC	S27^{o} 37’ 31.1”	W48^{o} 27’ 01.7”
Joaquina - SC	S27^{o} 37’ 35.5”	W48^{o} 26’ 59.7”

Joaquina - SC	S27^{o} 37’ 41.0”	W48^{o} 26’ 59.0”
Joaquina - SC	S27^{o} 37’ 45.2”	W48^{o} 27’ 03.4”
Joaquina - SC	S27^{o} 37’ 50.2”	W48^{o} 27’ 06.7”
Joaquina - SC	S27^{o} 37’ 53.0”	W48^{o} 27’ 08.1”
Joaquina - SC	S27^{o} 37’ 56.1”	W48^{o} 27’ 11.1”
Joaquina - SC	S27^{o} 38’ 01.1”	W48^{o} 27’ 18.3”
Joaquina - SC	S27^{o} 38’ 05.2”	W48^{o} 27’ 19.9”
Joaquina - SC	S27^{o} 38’ 10.3”	W48^{o} 27’ 23.9”
Pantano do Sul - SC	S27^{o} 46’ 52.9”	W48^{o} 31’ 12.8”
Pantano do Sul - SC	S27^{o} 46’ 51.7”	W48^{o} 31’ 09.1”
Pantano do Sul - SC	S27^{o} 46’ 49.2”	W48^{o} 31’ 03.6”
Pantano do Sul - SC	S27^{o} 46’ 49.5”	W48^{o} 31’ 01.5”
Pantano do Sul - SC	S27^{o} 46’ 48.3”	W48^{o} 30’ 56.3”
Torres - RS	S29^{o} 21’ 47.9”	W49^{o} 44’ 27.4”
Torres - RS	S29^{o} 21’ 51.8”	W49^{o} 44’ 31.7”
Torres - RS	S29^{o} 22’ 01.2”	W49^{o} 44’ 41.5”
Torres - RS	S29^{o} 22’ 00.3”	W49^{o} 44’ 43.1”
Torres - RS	S29^{o} 21’ 59.6”	W49^{o} 44’ 45.6”
Torres - RS	S29^{o} 21’ 58.3”	W49^{o} 44’ 48.9”
Torres - RS	S29^{o} 21’ 58.3”	W49^{o} 44’ 51.8”
Torres - RS	S29^{o} 21’ 59.1”	W49^{o} 44’ 55.3”
Torres - RS	S29^{o} 21’ 57.3”	W49^{o} 45’ 01.0”
Torres - RS	S29^{o} 22’ 02.8”	W49^{o} 45’ 01.9”
Curumin - RS	S29^{o} 37’ 11.7”	W49^{o} 55’ 54.7”
Curumin - RS	S29^{o} 37’ 09.0”	W49^{o} 55’ 52.7”
Curumin - RS	S29^{o} 37’ 04.4”	W49^{o} 55’ 50.3”
Curumin - RS	S29^{o} 37’ 00.8”	W49^{o} 55’ 49.8”
Curumin - RS	S29^{o} 36’ 59.5”	W49^{o} 55’ 50.7”
Curumin - RS	S29^{o} 36’ 58.5”	W49^{o} 55’ 49.1”
Curumin - RS	S29^{o} 36’ 58.2”	W49^{o} 55’ 45.9”
Curumin - RS	S29^{o} 36’ 53.9”	W49^{o} 55’ 43.8”
Curumin - RS	S29^{o} 36’ 51.3”	W49^{o} 55’ 41.6”
Curumin - RS	S29^{o} 37’ 18.1”	W49^{o} 55’ 59.5”
Xangrilá - RS	S29^{o} 49’ 02.3”	W50^{o} 02’ 33.9”
Xangrilá - RS	S29^{o} 48’ 59.8”	W50^{o} 02’ 33.1”
Xangrilá - RS	S29^{o} 48’ 58.0”	W50^{o} 02’ 32.9”
Xangrilá - RS	S29^{o} 49’ 21.9”	W50^{o} 02’ 45.3”
Xangrilá - RS	S29^{o} 49’ 23.3”	W50^{o} 02’ 45.9”
Cidreira - RS	S30^{o} 08’ 08.7”	W50^{o} 11’ 13.3”
Cidreira - RS	S30^{o} 07’ 58.1”	W50^{o} 11’ 10.8”
Cidreira - RS	S30^{o} 07’ 55.6”	W50^{o} 11’ 12.0”
Cidreira - RS	S30^{o} 07’ 49.1”	W50^{o} 11’ 08.0”
Quintão - RS	S30^{o} 23’ 41.4”	W50^{o} 18’ 01.8”
Quintão - RS	S30^{o} 23’ 39.2”	W50^{o} 18’ 01.1”
Quintão - RS	S30^{o} 23’ 34.9”	W50^{o} 17’ 57.3”
Quintão - RS	S30^{o} 23’ 48.2”	W50^{o} 18’ 03.8”

Quintão - RS	S30° 23’ 47.5”	W50° 18’ 08.8”
Quintão - RS	S30° 23’ 47.0”	W50° 18’ 13.0”
Quintão - RS	S30° 23’ 43.4”	W50° 18’ 15.0”
Mostardas - RS	S31° 07’ 43.4”	W50° 49’ 57.2”
Mostardas - RS	S31° 07’ 47.9”	W50° 49’ 54.3”
Mostardas - RS	S31° 07’ 48.2”	W50° 49’ 55.8”
Mostardas - RS	S31° 06’ 37.2”	W50° 52’ 13.4”
Mostardas - RS	S31° 06’ 37.2”	W50° 52’ 12.4”
Mostardas - RS	S31° 06’ 33.8”	W50° 52’ 36.4”
Mostardas - RS	S31° 06’ 32.1”	W50° 52’ 47.4”
Mostardas - RS	S31° 06’ 30.3”	W50° 52’ 58.4”
Mostardas - RS	S31° 06’ 22.6”	W50° 53’ 47.9”
São Simão - RS	S30° 58’ 51.2”	W50° 48’ 22.2”
São Simão - RS	S30° 56’ 04.4”	W50° 45’ 43.8”
Tavares - RS	S31° 19’ 12.7”	W51° 06’ 54.0”
Tavares - RS	S31° 19’ 11.3”	W51° 06’ 55.8”
Tavares - RS	S31° 19’ 16.7”	W51° 06’ 53.6”
Tavares - RS	S31° 19’ 18.2”	W51° 06’ 48.3”
Tavares - RS	S31° 19’ 17.1”	W51° 06’ 46.7”
Tavares - RS	S31° 19’ 16.5”	W51° 06’ 44.0”
Tavares - RS	S31° 19’ 15.7”	W51° 06’ 41.7”
Tavares - RS	S31° 19’ 16.6”	W51° 06’ 40.1”
Tavares - RS	S31° 19’ 19.9”	W51° 06’ 46.2”
Tavares - RS	S31° 19’ 20.8”	W51° 06’ 51.4”
São José do Norte - RS	S32° 01’ 39.8”	W52° 01’ 56.3”
São José do Norte - RS	S32° 01’ 39.8”	W52° 01’ 55.3”
São José do Norte - RS	S32° 01’ 37.7”	W52° 01’ 53.6”
São José do Norte - RS	S32° 01’ 38.9”	W52° 01’ 51.2”
São José do Norte - RS	S32° 01’ 33.9”	W52° 01’ 57.6”
São José do Norte - RS	S32° 01’ 37.6”	W52° 01’ 57.7”
São José do Norte – RS	S32° 01’ 37.2”	W52° 01’ 59.0”
São José do Norte – RS	S32° 01’ 37.7”	W52° 02’ 01.5”
São José do Norte – RS	S32° 01’ 39.2”	W52° 02’ 05.0”
São José do Norte - RS	S32° 01’ 41.9”	W52° 02’ 07.3”
Estreio - RS	S31° 51’ 11.0”	W51° 47’ 25.1”
Estreio - RS	S31° 51’ 08.9”	W51° 47’ 26.1”
Estreio - RS	S31° 51’ 08.1”	W51° 47’ 27.2”
Estreio - RS	S31° 51’ 06.0”	W51° 47’ 27.0”
Estreio - RS	S31° 51’ 04.1”	W51° 47’ 30.8”
Cassino - RS	S32° 13’ 19.8”	W52° 12’ 03.4”
Cassino - RS	S32° 06’ 57.3”	W52° 10’ 25.0”
Cassino - RS	S32° 06’ 57.7”	W52° 10’ 24.0”
Cassino - RS	S32° 06’ 57.2”	W52° 10’ 23.3”
Cassino - RS	S32° 06’ 55.8”	W52° 10’ 22.5”
Cassino - RS	S32° 06’ 55.3”	W52° 10’ 21.9”
Cassino - RS	S32° 06’ 52.1”	W52° 10’ 18.3”

Cassino - RS	S32° 06’ 52.6”	W52° 10’ 16.8”
Cassino - RS	S32° 06’ 51.8”	W52° 10’ 14.9”
Cassino - RS	S32° 06’ 50.0”	W52° 10’ 13.9”
Santa Maria - RS	S29° 39’ 11.7”	W54° 03’ 15.6”
Santa Maria - RS	S29° 39’ 11.7”	W54° 03’ 14.9”
Santa Maria - RS	S29° 39’ 10.5”	W54° 03’ 14.9”
Santa Maria - RS	S29° 39’ 05.9”	W54° 03’ 14.8”
Santa Maria - RS	S29° 39’ 04.9”	W54° 03’ 13.9”
Santa Maria - RS	S29° 39’ 05.1”	W54° 03’ 16.7”
Santa Maria - RS	S29° 39’ 05.9”	W54° 03’ 16.7”
Santa Maria - RS	S29° 39’ 07.3”	W54° 03’ 16.3”
Santa Maria - RS	S29° 39’ 06.9”	W54° 03’ 15.2”
Santa Maria - RS	S29° 39’ 08.2”	W54° 03’ 14.9”
Restinga Seca - RS	S29° 45’ 58.4”	W53° 29’ 26.3”
Restinga Seca - RS	S29° 46’ 01.2”	W53° 29’ 36.9”
Restinga Seca - RS	S29° 46’ 02.3”	W53° 29’ 39.5”
Restinga Seca - RS	S29° 46’ 02.7”	W53° 29’ 40.8”
Restinga Seca - RS	S29° 46’ 03.1”	W53° 29’ 38.7”
Santiago - RS	S29° 10’ 48.4”	W54° 50’ 59.3”
Santiago - RS	S29° 10’ 49.3”	W54° 51’ 00.4”
Santiago - RS	S29° 10’ 50.5”	W54° 50’ 57.9”
Santiago - RS	S29° 10’ 50.3”	W54° 50’ 56.3”
Santiago - RS	S29° 10’ 49.7”	W54° 50’ 57.7”
Unistalda - RS	S29° 02’ 22.6”	W55° 12’ 52.1”
Unistalda - RS	S29° 02’ 22.7”	W55° 12’ 52.1”
Unistalda - RS	S29° 02’ 22.6”	W55° 12’ 49.6”
Unistalda - RS	S29° 02’ 22.9”	W55° 12’ 50.2”
Unistalda - RS	S29° 02’ 22.7”	W55° 12’ 51.0”
Cruz Alta – RS	S28° 57’ 11.8”	W53° 38’ 49.4”
Cruz Alta – RS	S28° 57’ 11.1”	W53° 38’ 50.0”
Cruz Alta – RS	S28° 57’ 09.1”	W53° 38’ 50.3”
Cruz Alta – RS	S28° 57’ 08.2”	W53° 38’ 50.6”
Cruz Alta – RS	S28° 57’ 07.1”	W53° 38’ 50.7”
Cachoeira do Sul - RS	S29° 50’ 26.1”	W52° 57’ 57.7”
Cachoeira do Sul - RS	S29° 50’ 26.9”	W52° 57’ 57.2”
Cachoeira do Sul - RS	S29° 50’ 27.8”	W52° 57’ 59.2”
Cachoeira do Sul - RS	S29° 50’ 27.5”	W52° 58’ 01.8”
Cachoeira do Sul - RS	S29° 50’ 27.0”	W52° 58’ 02.9”
Cachoeira do Sul - RS	S29° 50’ 27.4”	W52° 58’ 04.5”
Cachoeira do Sul - RS	S29° 50’ 26.1”	W52° 58’ 03.0”
Cachoeira do Sul - RS	S29° 50’ 25.1”	W52° 58’ 02.3”
Caçapava do Sul - RS	S30° 36’ 44.7”	W53° 21’ 35.2”
Caçapava do Sul - RS	S30° 36’ 44.2”	W53° 21’ 36.1”
Caçapava do Sul - RS	S30° 36’ 45.2”	W53° 21’ 37.6”
Caçapava do Sul - RS	S30° 36’ 45.8”	W53° 21’ 38.1”
Caçapava do Sul - RS	S30° 36’ 43.8”	W53° 21’ 36.5”

Pedro Osório - RS	S32º 01’ 07.1”	W52º 49’ 48.9”
Pedro Osório - RS	S32º 01’ 06.5”	W52º 49’ 47.7”
Pedro Osório - RS	S32º 01’ 05.2”	W52º 49’ 46.1”
Pedro Osório - RS	S32º 00’ 29.7”	W52º 49’ 18.1”
Bagé - RS	S31º 22’ 05.9”	W54º 07’ 03.0”
Bagé - RS	S31º 22’ 05.2”	W54º 07’ 04.6”
Bagé - RS	S31º 22’ 05.0”	W54º 07’ 05.4”
Bagé - RS	S31º 22’ 04.7”	W54º 07’ 06.0”
Bagé - RS	S31º 22’ 06.2”	W54º 07’ 02.2”
Bagé - RS	S31º 22’ 05.4”	W54º 06’ 58.1”
Bagé - RS	S31º 22’ 05.9”	W54º 06’ 51.2”
Tapes - RS	S30º 39’ 08.6”	W51º 33’ 54.1”
Tapes - RS	S30º 39’ 08.5”	W51º 33’ 57.9”
Tapes - RS	S30º 39’ 09.4”	W51º 33’ 56.1”
Tapes - RS	S30º 39’ 10.2”	W51º 33’ 53.5”
Tapes - RS	S30º 39’ 09.3”	W51º 33’ 50.5”
Santa Rosa - Argentina	S36º 37’ 07.1”	W64º 19’ 42.8”
Santa Rosa - Argentina	S36º 37’ 06.8”	W64º 19’ 42.9”
Santa Rosa - Argentina	S36º 37’ 06.7”	W64º 19’ 43.5”
Santa Rosa - Argentina	S36º 37’ 06.5”	W64º 19’ 42.4”
Santa Rosa - Argentina	S36º 37’ 05.8”	W64º 19’ 42.6”
Ataliba Roca - Argentina	S37º 03’ 00.9”	W64º 17’ 05.3”
Ataliba Roca - Argentina	S37º 03’ 00.2”	W64º 17’ 04.9”
Ataliba Roca - Argentina	S37º 03’ 00.1”	W64º 17’ 05.3”
Ataliba Roca - Argentina	S37 º 02’ 59.7”	W64º 17’ 05.2”
Ataliba Roca - Argentina	S37º 03’ 00.6”	W64º 17’ 05.9”
Ataliba Roca - Argentina	S37º 03’ 00.0”	W64º 17’ 06.0”
Entre Lihue Calel - Argentina	S37º 32’ 03.4”	W65º 09’ 20.1”
Entre Lihue Calel - Argentina	S37º 32’ 04.7”	W65º 09’ 21.2”
Entre Lihue Calel - Argentina	S37º 32’ 01.7”	W65º 09’ 18.0”
Entre Lihue Calel - Argentina	S37º 32’ 01.6”	W65º 09’ 17.9”
Entre Lihue Calel - Argentina	S37º 32’ 01.0”	W65º 09’ 19.4”
Parque Lihue Calel - Argentina	S37º 52’ 56.5”	W65º 27’ 56.0”
Parque Lihue Calel - Argentina	S37º 52’ 57.6”	W65º 27’ 57.0”
Parque Lihue Calel - Argentina	S37º 52’ 59.0”	W65º 27’ 58.2”
Parque Lihue Calel - Argentina	S37º 52’ 54.9”	W65º 27’ 54.5”
Winifreda - Argentina	S36º 12’ 34.4”	W64º 16’ 39.3”
Winifreda - Argentina	S36º 12’ 34.9”	W64º 16’ 39.4”
Winifreda – Argentina	S36º 12’ 34.6”	W64º 16’ 39.9”
Winifreda - Argentina	S35º 13’ 31.4”	W64º 15’ 54.2”

SC: Santa Catarina State; RS: Rio Grande do Sul State.
