# Supplementary material for: Integrating Paleodistribution Models and Phylogeography in the Grass-Cutting Ant Acromyrmex striatus (Hymenoptera: Formicidae) in Southern Lowlands of South America
Source: PLoS One. 2016 Jan 6;11(1):e0146734. doi: 10.1371/journal.pone.0146734 (PMC4703384; doi:10.1371/journal.pone.0146734)
Supplement: S2 Table — (PDF) [file pone.0146734.s004.pdf]

Table S2 - Analysis of molecular variance (AMOVA) for the *A. striatus*, performed with three hierarchical levels, considering each 38 sites in Brazil e Argentina (populations), considering the groups formed by SAMOVA (7 groups).

**Source of variation**	**d.f.**	**Percentage of variation**
Among groups	6	79.46
Among populations within groups	31	7.73
Within groups	90	12.82
