# Supplementary material for: Integrating Paleodistribution Models and Phylogeography in the Grass-Cutting Ant Acromyrmex striatus (Hymenoptera: Formicidae) in Southern Lowlands of South America
Source: PLoS One. 2016 Jan 6;11(1):e0146734. doi: 10.1371/journal.pone.0146734 (PMC4703384; doi:10.1371/journal.pone.0146734)
Supplement: S3 Table — Values shown in bold are significant (p < 0.05). (PDF) [file pone.0146734.s005.pdf]

Table S3 – F_{ST} values of pair-wise comparisons between populations of *Acromyrmex striatus*, estimated from the mitochondrial COI gene. Values shown in bold are significant ($p < 0.05$).

	FLOR	CONT	CABO	LAGU	SOUT	EXTR	NORT	MOTD	RIOG	INTE	SAMA	WEST	ARGE
FLOR	0.0000												
CONT	0.2871	0.0000											
CABO	0.1140	0.0903	0.0000										
LAGU	0.0000	0.0222	0.0134	0.0000									
SOUT	0.0318	0.0418	-0.0112	-0.0251	0.0000								
EXTR	0.0811	0.0387	0.1030	0.0165	0.0192	0.0000							
NORT	**0.5808**	**0.5145**	**0.5160**	**0.5369**	**0.5370**	**0.5652**	0.0000						
MOTD	**0.9023**	**0.8758**	**0.8460**	**0.8513**	**0.8473**	**0.8712**	**0.3625**	0.0000					
RIOG	**0.8703**	**0.8442**	**0.8244**	**0.8306**	**0.8284**	**0.8451**	**0.6427**	**0.8475**	0.0000				
INTE	**0.7765**	**0.7349**	**0.7189**	**0.7270**	**0.7264**	**0.7458**	**0.3622**	**0.5283**	**0.7930**	0.0000			
SAMA	**0.8790**	**0.8429**	**0.8133**	**0.8221**	**0.8177**	**0.8400**	**0.3989**	**0.6719**	**0.8301**	**0.1276**	0.0000		
WEST	**0.9376**	**0.9163**	**0.8815**	**0.8833**	**0.8769**	**0.8977**	**0.4999**	**0.7271**	**0.8702**	0.0405	**0.2810**	0.0000	
ARGE	**0.9494**	**0.9297**	**0.9128**	**0.9195**	**0.9179**	**0.9296**	**0.7090**	**0.9103**	**0.8805**	**0.8585**	**0.8872**	**0.9289**	0.000
